# Epigenetic Silencing of Ubiquitin Specific Protease 4 by Snail1 Contributes to Macrophage-Dependent Inflammation and Therapeutic Resistance in Lung Cancer

**DOI:** 10.3390/cancers12010148

**Published:** 2020-01-08

**Authors:** Chao-Yang Lai, Da-Wei Yeh, Chih-Hao Lu, Yi-Ling Liu, Yu-Chen Chuang, Jhen-Wei Ruan, Cheng-Yuan Kao, Li-Rung Huang, Tsung-Hsien Chuang

**Affiliations:** 1 Immunology Research Center, National Health Research Institutes, Zhunan, Miaoli County 35053, Taiwan; chaoyang@nhri.org.tw (C.-Y.L.); daweiyeh@mail2000.com.tw (D.-W.Y.); jamal770903@gmail.com (C.-H.L.); lil5410@nhri.org.tw (Y.-L.L.); yuchenc@nhri.edu.tw (Y.-C.C.); jhenweiruan@mail.ncku.edu.tw (J.-W.R.); chengyuankao@nhri.edu.tw (C.-Y.K.); 2 Institute of Molecular and Genomic Medicine, National Health Research Institutes, Zhunan, Miaoli County 35053, Taiwan; 020401@nhri.org.tw; 3 Program in Environmental and Occupational Medicine, Kaohsiung Medical University, Kaohsiung City 80708, Taiwan

**Keywords:** deubiquitination, Snail1, inflammation, stemness, epigenetic regulation

## Abstract

There is a positive feedback loop driving tumorigenesis and tumor growth through coordinated regulation of epigenetics, inflammation, and stemness. Nevertheless, the molecular mechanism linking these processes is not well understood. In this study, we analyzed the correlation of de-ubiquitinases (DUBs) expression with survival data from the OncoLnc database. Among the DUBs analyzed, ubiquitin specific protease 4 (USP4) had the lowest negative Cox coefficient. Low expression of USP4 was associated with poor survival among lung cancer patients and was inversely correlated with expression of stemness and inflammation markers. Expression of USP4 were reduced at more advanced stages of lung cancer. Mechanistically, expression of USP4 was downregulated in snail1-overexpressing and stemness-enriched lung cancer cells. Snail1 was induced in lung cancer cells by interaction with macrophages, and epigenetically suppressed USP4 expression by promoter methylation. Stable knockdown of USP4 in lung cancer cells enhanced inflammatory responses, stemness properties, chemotherapy resistance, and the expression of molecules allowing escape from immunosurveillance. Further, mice injected with USP4 knockdown lung cancer cells demonstrated enhanced tumorigenesis and tumor growth. These results reveal that the Snail1-mediated suppression of USP4 is a potential mechanism to orchestrate epigenetic regulation, inflammation and stemness for macrophage-promoted tumor progression.

## 1. Introduction

Lung cancer is one of the most mortal cancers worldwide. Despite extensive research efforts to improve diagnosis and treatment, more than 50% of lung cancer patients die within one year of diagnosis, and the 5-year survival rate is lower than 18% [1,2]. Inflammation is a hallmark of tumor development, and roughly 20–25% of risk factors are related to inflammation [3,4]. Chronic inflammation resulting from viral infections, pneumonia, tuberculosis, and chronic obstructive pulmonary disease is associated with lung cancer development [5,6]. The transcription factor NF-κB is the master regulator of inflammation [7,8]. Major inflammatory stimuli in the tumor microenvironment such as tumor necrosis factor (TNF)-α, interleukin (IL)-1, and toll-like receptor (TLR) ligands released from dying cells during cancer treatment activate NF-κB in surviving cancer cells [9,10,11]. Activation of the IL-1 receptor (IL-1R) and TLRs triggers the sequential recruitment of MyD88, IRAK, and TRAF6 to form a complex to activate TAK, which in turn leads to NF-κB activation [12,13]. Alternatively, NF-κB activation by the TNF-α receptor (TNFR) involves the signaling molecules TRADD, RIP, and TRAF2 [14]. Ensuing NF-κB activation regulates expression of multiple genes that can support tumor development through suppression of apoptosis, enhanced angiogenesis, and promotion of cancer cell proliferation, migration, and invasion [7,15,16].

Metastasis and recurrence are the major challenges in lung cancer treatment, and both are associated with epithelial−mesenchymal transition (EMT) and acquisition of stemness in cancer cells. EMT is a process by which epithelial cells lose their epithelial properties and gain the characteristics of mesenchymal cells. In cancer development, EMT is regarded as an initial step for metastasis. In this process, cancer cells lose cell−cell adhesion properties and gain migration and invasion capacities. In addition, EMT has been shown to confer stemness properties to cancer cells [17,18]. Cancer stem-like cells (CSCs) have capacities for both self-renewal and differentiation to promote tumor progression and metastasis. Moreover, CSCs are less immunogenic, and so can evade immune surveillance and ensuing destruction. These properties are responsible for cancer treatment resistance and relapse [19,20], and NF-κB-mediated inflammation elevates the stemness properties of cancer cells. Moreover, CSCs are known to exhibit higher constitutive NF-κB activity. Thus, these two bidirectional effects form a positive feedback loop to further expand the CSC population in tumors, resulting in therapeutic resistance and poor prognosis [21,22].

Dysregulation of genes involved in the inflammation and stemness properties of cancer cells can lead to tumorigenesis and tumor progression [21,22]. Epigenetic regulation by promoter methylation alters gene expression at the transcriptional level, and altered epigenomic characteristics are associated with tumor inflammation and stemness, although the underlying mechanisms are not well understood [23,24]. Snail1 is a member of the Snail family of zinc-finger transcription factors, which also includes Snail2 (Slug) and Snail3 (Smuc). Snail1 plays a key role in regulation of EMT and dedifferentiation of cancer cells into CSCs, at least in part by epigenetic silencing of E-cadherin gene expression, resulting in reduced cell adhesion and increased cell migration. In this process, Snail1 recruits multiple chromatin enzymes to the E-cadherin promoter in a highly orchestrated process to form heterochromatin and facilitate DNA methylation of promoter DNA. Snail1 is expressed in many different types of cancers and correlates with increased invasion and metastasis [25,26,27,28,29].

Ubiquitination is a multistep post-transcriptional modification process in which mono-ubiquitin or poly-ubiquitin chains are attached to substrate protein. Ubiquitin contains 76 amino acid residues of which 7 are lysines. A ubiquitin chain can be linked to a target protein through lysine residues at different positions to determine the fate of the target protein. For example, a ubiquitin linked through lysine 48 (K48) targets the protein for proteasomal degradation and an ubiquitin chain linked through lysine 63 (K63) mediates protein−protein interactions for signal transduction [30,31]. The ubiquitination process can be reversed by deubiquitinating enzymes (DUBs) that cleave ubiquitin off the substrate protein. There are approximately 100 DUBs encoded in the human genome. An individual DUB can be a positive or negative regulator of a specific signaling pathway depending on target protein and type of ubiquitination [32,33]. For example, ubiquitin specific protease (USP)4 has been shown to act as a negative regulator of TNFR-, IL-1R-, and TLR-mediated NF-κB activation and inflammatory responses through removal of ubiquitin chains from signaling molecules, including TGF-β-activated kinase 1 (TAK1), receptor-interacting protein (RIP1), TNF receptor-associated factor (TRAF)2, and TRAF6, thereby preventing protein-protein interactions for signaling transduction [34,35,36].

These DUBs are critical for maintaining multiple cellular functions, and dysregulation of DUBs can result in numerous clinical disorders [37,38]. In this study, we analyzed an OncoLnc database for correlations between lung cancer patient survival and the expression levels of different DUBs. Among DUBs analyzed, USP4 had the lowest negative Cox coefficient, suggesting a beneficial role of higher USP4 expression on lung cancer outcome. Further, low expression of USP4 was associated with poor prognosis. Thus, the mechanism for downregulation of USP4 and the functions of this DUB in control of inflammation, stemness, and lung cancer growth were further investigated experimentally both in culture and in mouse models of tumorigenesis and tumor growth.

## 2. Results

### 2.1. Downregulation of USP4 in Lung Cancer Is Associated with Poor Prognosis and High Expression of Stemness and Inflammation Markers

**Figure 1:**
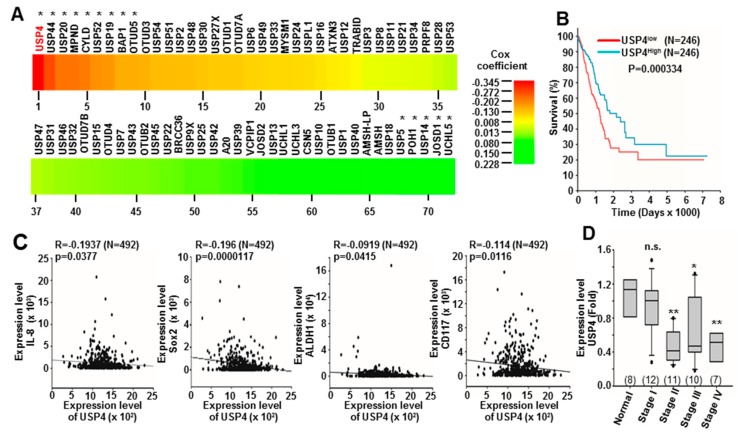
Inverse correlations of ubiquitin specific protease 4 (USP4) expression with stemness markers, inflammation markers, and lung cancer prognosis. (**A**) Heat map of Cox coefficients for 72 deubiquitinase (DUB) genes according to OncoLnc database analysis. (**B**) Kaplan-Meier plot of the overall survival rate of lung cancer patients stratified by low (n = 246) and high (n = 246) USP4 expression (from OncoLnc). (**C**) Inverse correlations between USP4 expression level and the expression levels of various inflammation and stemness markers (from OncoLnc). Sample numbers are shown in parentheses. (**D**) Relative expression levels of USP4 in normal tissues and different stages of human lung cancer. Patient data are summarized in Table S2. Expression of USP4 was analyzed by qPCR. Numbers in parentheses represent sample numbers for each cancer stage. * *P* < 0.05; ** *P* < 0.01.

OncoLnc contains survival data of more than 8000 patients from 21 cancer studies performed by The Cancer Genome Atlas, and provides a tool for searching correlations between survival and the expression levels of various mRNAs, miRNAs, and lncRNAs [39]. To identify DUBs that may contribute to lung cancer development or suppression, we searched OncoLnc for the survival data of lung adenocarcinoma patients with measured DUB expression. The Cox coefficients of 72 DUBs were retrieved by this search, of which USP4 had a highest negative Cox coefficient (Figure 1A and Table S1). 

This negative correlation suggests that USP4 expression is beneficial to lung cancer patient survival. These OncoLnc data were further stratified into high (top 50%) and low (bottom 50%) USP4 expression subgroups, and subgroup survival compared by Kaplan-Meier analysis. The low expression subgroup demonstrated shorter overall survival compared to the high expression subgroup (Figure 1B), indicating that low USP4 expression is associated with poor lung cancer prognosis. Correlations between the expression levels of USP4 and various inflammation and stemness markers were also analyzed from OncoLnc data. Low expression of USP4 was associated with high expression of the pro-inflammatory cytokine IL-8 as well as with upregulation of the stemness markers Sox2, ALDH1, and CD117 (Figure 1C).

The expression levels of USP4 in tissues of normal and different cancer stages were then examined by qPCR using an array with 48 cDNA samples from lung cancer patients (clinical data summarized in Table S2). Consistent with OncoLnc results, the expression level of USP4 was significantly reduced in stage II to stage IV lung cancer tissues compared to normal human lung tissue (Figure 1D). USP4 expression levels in various normal and cancerous tissue types were further investigated by analysis of data from Oncomine, which revealed lower USP4 expression in multiple head and neck, breast, and lung cancers compared to matched normal tissues (Figure S1). Further analysis of data from the GEO database also revealed that USP4 expression was downregulated in different head and neck, breast, and lung cancer cells following enhancement of stemness by sphere formation, Bmi1 and Snail overexpression, or chemotherapeutic treatments (Table S3).

### 2.2. Downregulation of USP4 in Stemness-Enriched Cancer Cells 

**Figure 2:**
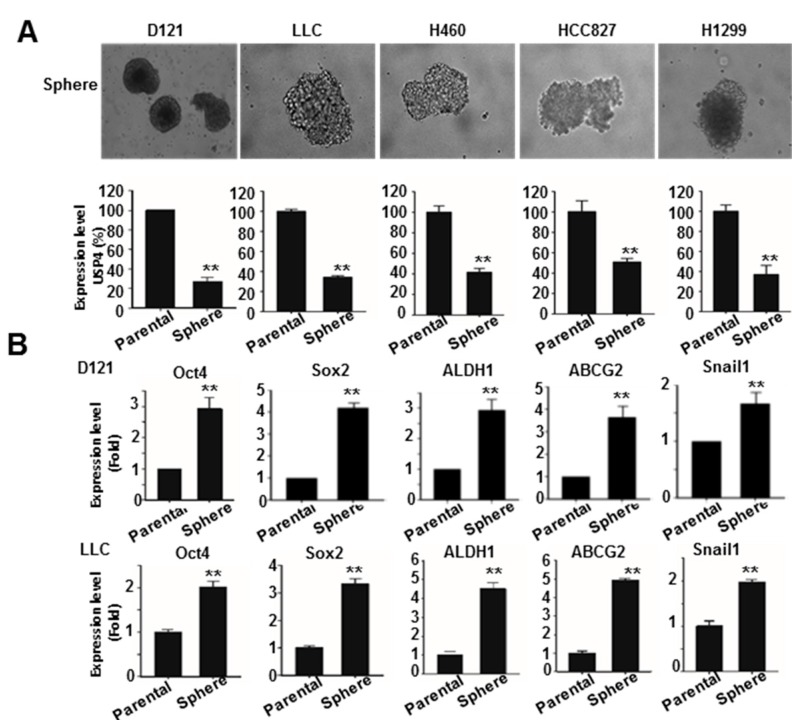
Downregulation of USP4 in stemness-enriched lung cancer cells. (**A**) Top panels: Stemness of mouse D121, LLC, and human H460, HCC827, and H1299 lung cancer cell lines was enriched by sphere formation. Photos show sphere cells of each cell line. (**B**) Bottom panels: Expression of stemness-associated genes in parental D121 and LLC cells and the corresponding sphere cells analyzed by RT-qPCR. Data presented as mean ± SD of three independent experiments. ** *P* < 0.01.

The effect of stemness on USP4 expression was further investigated. The stemness of lung cancer cell lines (mouse D121, Lewis lung carcinoma (LLC), and human H460, HCC827, and H1299) was enriched by sphere formation. Gene expression analysis by RT-qPCR demonstrated lower USP4 expression in sphere cells than the parental cells for each line (Figure 2A). The expression levels of USP4 and different stemness-associated genes were then compared between parental D121 and LLC cells and corresponding sphere-forming cells RT-qPCR (Figure 2B), which indicated increased expression levels of stemness-associated genes Oct4, Sox2, ALDH1, ABCG2, and Snail1 in the sphere cells, while USP4 expression was reduced in spheroid cells compared to the parental cells (Figure 2B). 

These results are consistent with the results of OncoLnc database analysis (Figure 1C) showing inverse correlations between expression levels of USP4 and different stemness markers as well as with the results of GEO database analysis demonstrating lower USP4 expression in stemness-enriched cells (Table S3).

### 2.3. Snail1 Promotes DNA Methylation of the USP4 Promoter and Suppresses USP4 Expression 

**Figure 3:**
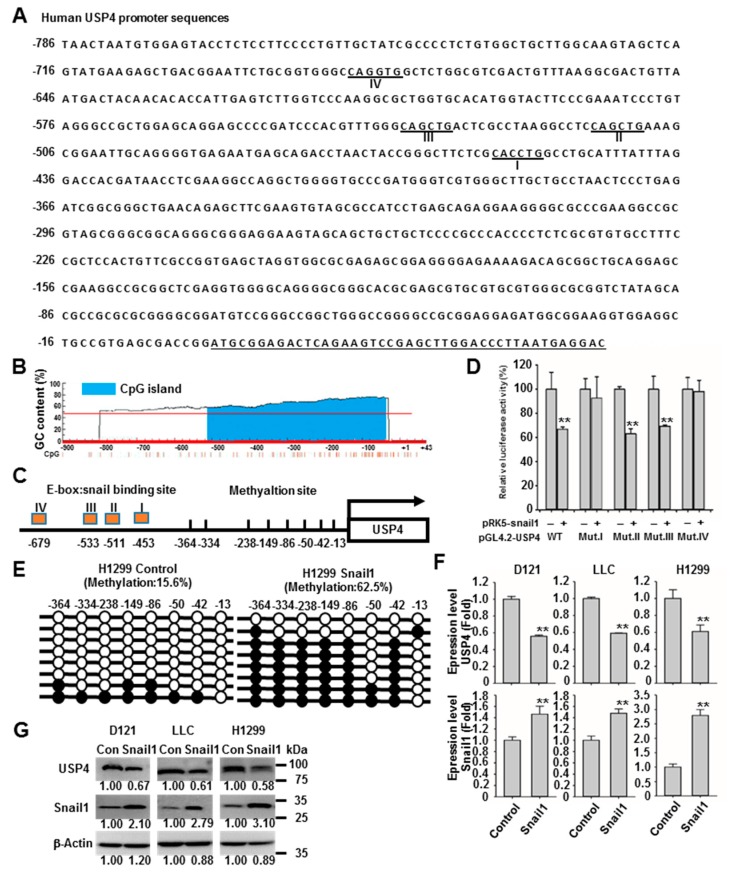
Snail1 enhances DNA methylation of the USP4 promoter and suppresses USP4 expression. (**A**) Promoter region of USP4 was analyzed for Snail1 binding sites. The putative binding sites are underlined. (**B**) Promoter region of USP4 was analyzed for GC rich (or CpG-rich) regions (potential methylation sites). The histogram was adopted from the MethPrimer website. (**C**) Illustration of Snail1 binding sites and methylation regions in the USP4 promoter region. (**D**) The binding sites of the USP4 promoter required for transcriptional suppression by Sanil1 were identified by luciferase assay. HEK293 cells were co-transfected with expression vectors for Snail1 and a luciferase reporter gene controlled by wild type (wt) mutated USP4 promoter sites. (**E**) DNA methylation of the USP4 promoter region in the presence and absence of Snail1 as determined by bisulfite sequencing PCR. (**F**,**G**) Expression levels of USP4 mRNA (F) and protein (G) in snail1-overexpressing D121, LLC, and H1299 cells. Data presented as mean ± SD of three independent experiments (E,F). ** *P* < 0.01.

**Figure 4:**
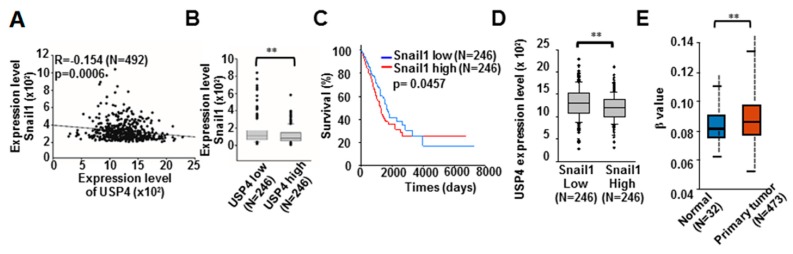
Inverse correlation of ubiquitin specific protease 4 (USP4) expression with Snail1 and methylation of USP4 promoter in lung cancers. OncoLnc data of lung cancer patients analyzed. (**A**) Inverse correlation between USP4 and Sanil1 expression levels. (**B**) Snail1 expression compared between subgroups stratified according to low and high USP4 expression. (**C**) Kaplan-Meier plot of the overall survival rate of lung cancer patients stratified by low (n = 246) and high (n = 246) Snail1 expression. (**D**) USP4 expression compared between subgroups of low and high USP4 expression. (**E**) Comparison of USP4 promoter DNA methylation level between normal tissue samples and lung tumors analyzed using UALCAN. ** *P* < 0.01.

Of these stemness-associated genes, Snail1 is known to function in epigenetic suppression of gene expression by binding to the promoter E-box motif 5’-CANNTG-3’ and orchestrating the activities of CpG island hypermethylation [25,26,27,28,29]. Thus, we investigated whether Snail1 also contributes to the reduction of USP4 expression in lung cancer cells. First, we confirmed the presence 4 E-boxes in the USP4 promoter at −453, −511, −533, and −679, as well as eight CpG-dinucleotides at (cytosine position) −13, −42, −50, −86, −149, −238, −334, and −364 (Figure 3A–C). To investigate if Snail1 can indeed bind and regulate the USP4 promoter, luciferase reporter constructs were generated in which luciferase expression was controlled by the wild type USP4 promoter or promoters with the E-box sequence mutations shown in Table S4 and co-transfected with Snail1 into HEK293 cells. Analysis of the generated luciferase activities revealed that expression of Snail1 suppressed USP4 promoter activity and that both E-box I and E-box IV were required for Snail1 activity at the USP4 promoter (Figure 3D). The methylation status of the eight CpG-dinucleotides was investigated using bisulfite sequencing PCR. Relative to control H1299 cells, cells stably overexpressing Snail1 exhibited greater DNA methylation of these CpG-dinucleotides (Figure 3E). Subsequently, we assessed the regulation of USP4 mRNA and protein expression by Snail1. Consistent with epigenetic suppression by hypermethylation of the USP4 promoter, overexpression of Snail1 in D121, LLC, and H1299 cells reduced USP4 expression at both the mRNA and protein levels (Figure 3F,G). The relationship between Snail1 and USP4 expression in lung cancer was further investigated through OncoLnc analysis, which revealed an association between high Snail1 and low USP4 expression (Figure 4A). There was also a significant difference in Snail1 expression between USP4 low (bottom 50%) and USP4 high (top 50%) lung cancer subgroups (Figure 4B). These OncoLnc data were further stratified into high (top 50%) and low (bottom 50%) Snail1 expression subgroups. The high Snail1 expression subgroup had shorter survival compared to the low expression subgroup in the first 3000 days (Figure 4C), indicating that low USP4 expression is associated with poor lung cancer prognosis. Consistently, there was a significant high and low USP4 expression in the Snail1 low and high groups respectively (Figure 4D). In addition, database searches for promoter DNA methylation status using the UALCAN program [40], revealed significantly higher methylation of the USP4 promoter in primary lung tumor compared to normal lung tissues (Figure 4E).

### 2.4. Macrophages Promote Snail1 Expression and USP4 Downregulation in Lung Cancer Cells

**Figure 5:**
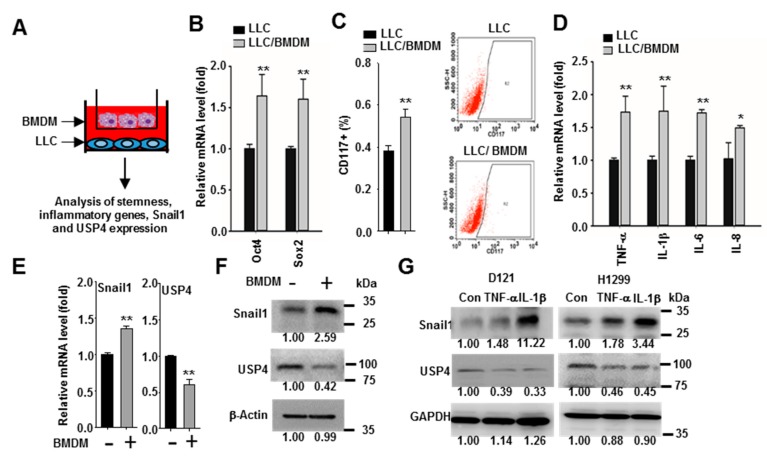
Macrophages promote inflammation, stemness, and Snail1 expression, and downregulates USP4 in lung cancer cells. (**A**) LLC lung cancer cells were co-cultured with or without bone marrow-derived macrophages (BMDMs) in 0.4 μm transwell plates as illustrated. (**B**) Expression levels of stemness-associated genes were analyzed by RT-qPCR. (**C**) Cell surface stemness marker CD117 analyzed by flow cytometry. Left panels show a set of the histograms. (**D**) Expression levels of inflammatory cytokines analyzed by RT-qPCR. (**E**,**F**) Expression of Snail1 and USP4 at the mRNA level (**E**) and protein level (**F**) analyzed with RT-qPCR and immunoblotting, respectively. (**G**) Inflammatory cytokines upregulate Snail1 and downregulate USP4 in lung cancer cells. D121 and H1299 were treated with TNF-α (20 ng/mL) or IL-1β (20 ng/mL) for 48 h. Data presented as mean ± SD of three independent experiments. * *P* < 0.05; ** *P* < 0.01.

Infiltration of immune cells into the tumor microenvironment is associated with cancer stemness and progression [41]. Macrophages are a major population of leukocytes that produce various cytokines in the tumor microenvironment [5,6,42,43] and expression of Snail1 is regulated by a wide variety of cytokines [25,26]. Therefore, we investigated whether macrophages regulate the expression of Snail1 in lung cancer cells. The LLC lung cancer line was co-cultured with bone marrow-derived macrophages (BMDMs) in two-chamber transwell plates (Figure 5A), and the gene expression profile compared to LLC monocultures was analyzed by RT-qPCR and flow cytometry analysis. These comparisons revealed greater expression of cytosolic stemness markers (Oct4 and Sox2) by co-cultured LCCs and a larger proportion expressing the surface stemness marker CD117 compared to LCC monocultures (Figure 5B,C). Further, these changes were associated with increased expression levels of the inflammatory cytokines TNF-α, IL-1β, IL-6, and IL-8 (Figure 5D). In addition, RT-qPCR and immunoblotting revealed that the presence of macrophages enhanced Snail1 expression and reduced USP4 expression in LCC cells at both the mRNA and protein levels (Figure 5E,F). We then examined the direct effects of inflammatory cytokines, which can be produced by macrophages, on USP4 and Snail1 expression levels. In line with macrophage co-culture results, both TNF-α and IL-1β treatment downregulated USP4 and upregulated Snail1 in D121 and H1299 cell lines as evidenced by immunoblot analysis (Figure 5G).

### 2.5. Downregulation of USP4 Increases Basal and Stimulus-Induced Expression of Pro-Inflammatory Factors by Cancer Cells 

**Figure 6:**
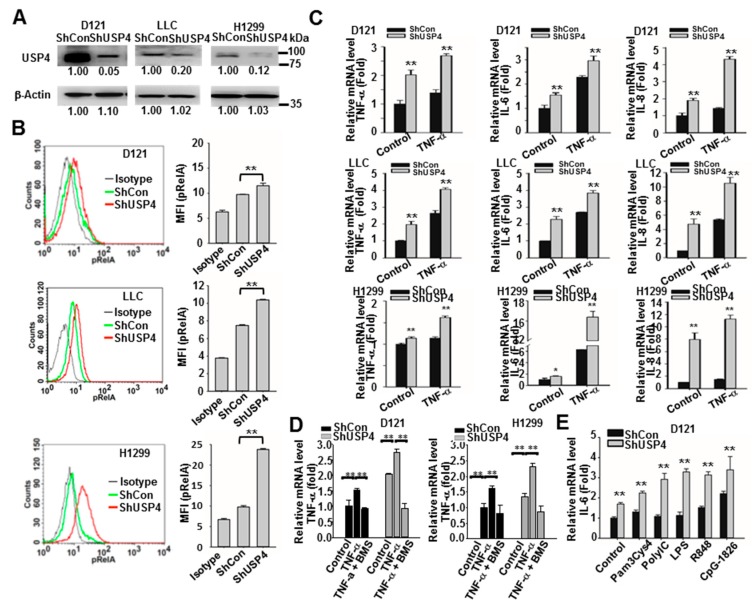
Stable USP4 knockdown increases inflammatory status in lung cancer cells. (**A**) Efficiency of USP4 knockdown in lung cancer cell lines analyzed by immunoblotting. (**B**) NF-κB activation in control and USP4 knockdown lung cancer cells as measured by flow cytometric analysis of RelA phosphorylation. Left panels show representative histograms and right panels shows the quantitation of results. (**C**–**E**) Expression of cytokines in control and USP4 knockdown lung cancer cells stimulated with 10 ng/mL TNF-α with/without 1 μM BMS345541 (C,D) or 0.2 μg/mL Pam3Cys4, 5 μg/mL polyI:C, 0.2 μg/mL LPS, 2 μM R847, or CpG-1826 (**E**) for 24 h as analyzed by RT-qPCR. Data presented as mean ± SD of three independent experiments. * *P* < 0.05; ** *P* < 0.01.

The functions of USP4 in regulation of cancer cell inflammatory status were then investigated. Expression of IL-8 is known to be controlled by NF-κB activation [44,45]. Database analysis revealed an inverse correlation between USP4 and IL-8 expression levels (Figure 1C), suggesting that USP4 may negatively regulate inflammatory responses in cancer cells. To examine this issue, USP4 expression in D121, LLC, and H1299 lung cancer cells was knocked down by stable transfection of a USP4-targeted shRNA (Figure 6A). Phosphorylation of RelA was measured by flow cytometry as an indicator of NF-κB activation. In all cancer cell lines, knockdown of USP4 increased intrinsic activation of NF-κB (Figure 6B). Moreover, knockdown of USP4 enhanced both basal expression levels of the NF-κB-controlled inflammatory cytokines TNF-α, IL-6, and IL-8 as well as induction of these cytokines by TNF-α and TLR ligands (pam3Cys4 for TLR2, polyIC for TLR3, LPS for TLR4, R848 for TLR7, and CpG-1826 for TLR9) (Figure 6C,E) as evidenced by RT-qPCR analysis. The cytokine inducing capability of TNF-α and the enhancement effect of USP4 knockdown were blocked by treatment of the cells with a NF-κB inhibitor, BMS345541 (Figure 6D). These findings suggest that USP4 is a negative regulator of cancer cell inflammatory status.

### 2.6. Downregulation of USP4 Promotes the Stemness and Therapeutic Resistance of Cancer Cells 

**Figure 7:**
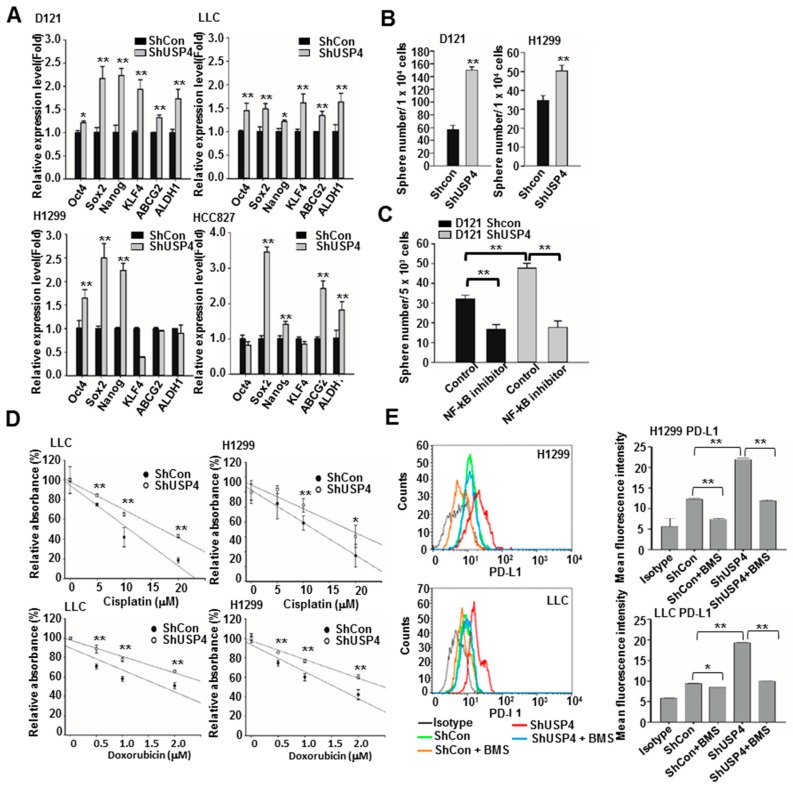
Stable USP4 knockdown increases the stemness, transforming ability, and therapeutic resistance of lung cancer cells. (**A**) Expression of stemness-associated genes in control and USP4 knockdown lung cancer cells analyzed by RT-qPCR. (**B**) Control and USP4 knockdown D121 and H1299 cells were cultured in defined serum-free medium for 2 weeks to allow sphere formation. (**C**) Sphere formation compared between control D121 cells and D121 cells with stable USP4 knockdown cultured in defined serum-free medium and treated with/without 1 μM BMS345541(NF-κB inhibitor) for 2 weeks. (**D**) Control and USP4 knockdown lung cancer cells were treated with the indicated concentration of cisplatin or doxorubicin for 48 h and cell viability measured by MTS assay. (**E**) Control and USP4 knockdown lung cancer cells were treated with/without 1 μM BMS345541 for 24 h. Cell surface expression of PD-L1 as analyzed by flow cytometry. Left panels: Representative histograms. Right panels: Quantitation. Data presented as mean ± SD of three independent experiments. * *P* < 0.05; ** *P* < 0.01.

According to database analysis, expression of USP4 was inversely correlated with expression of stemness markers in lung cancers (Figure 1C). In addition, the USP4 gene was downregulated during enrichment of stemness in cancer cells as evidenced by the sphere forming assays (Figure 2). Thus, we investigated whether USP4 directly regulates stemness. Indeed, knockdown of USP4 in the cancer cell lines D121, LLC, H1299, and HCC827 increased expression of the stemness genes Oct4, Sox2, Nanog, KLF4, ABCG2, and ALDH1 in different degree in different cell lines as measured by RT-qPCR (Figure 7A). Knockdown of USP4 expression also increased sphere formation by D121 and H1299 cells (Figure 7B). This increased propensity for sphere formation was NF-κB dependent as it was markedly reduced by pharmacological NF-κB inhibition (Figure 7C). The effect of USP4 downregulation on the transforming capacity of lung cancer cells was further investigated using cell proliferation and anchorage-independent growth assays. 

Downregulation of USP4 increased the proliferation rates of all cell lines examined after 48 h (Figure S2) as well as the number of colonies produced by these lines in soft agar after 3 weeks (Figure S3), indicating that USP4 downregulation enhances both the proliferative and transformation capacities of lung cancer cells.

Database searches revealed higher expression of USP4 in therapy-resistant lung tumors (Table S3); therefore, we further investigated whether downregulation of USP4 in lung cancer cells confers chemotherapy and immunotherapy resistance. Consistent with this notion, USP4 knockdown LLC and H1299 cells were more resistant to the cytotoxic effect of cisplatin and doxorubicin treatment for 48 h than corresponding control cells (Figure 7D). Cancer cells express programmed death-ligand 1 (PD-L1), which helps these cells to escape immunosurveillance by binding to PD-1 and reducing T cell activation [46,47]. Both LLC and H1299 cells with USP4 knockdown demonstrated greater surface expression of PD-L1 as analyzed by flow cytometry. Moreover, PD-L1 expression was reduced by treatment with the NF-κB inhibitor BMS345541 (Figure 7E). These results suggest that downregulation of USP4 enhances chemoresistance and helps lung cancer cells to evade destruction by anti-tumor immunity.

### 2.7. Downregulation of USP4 Promotes Tumorigenesis and Tumor Growth in Mice 

**Figure 8:**
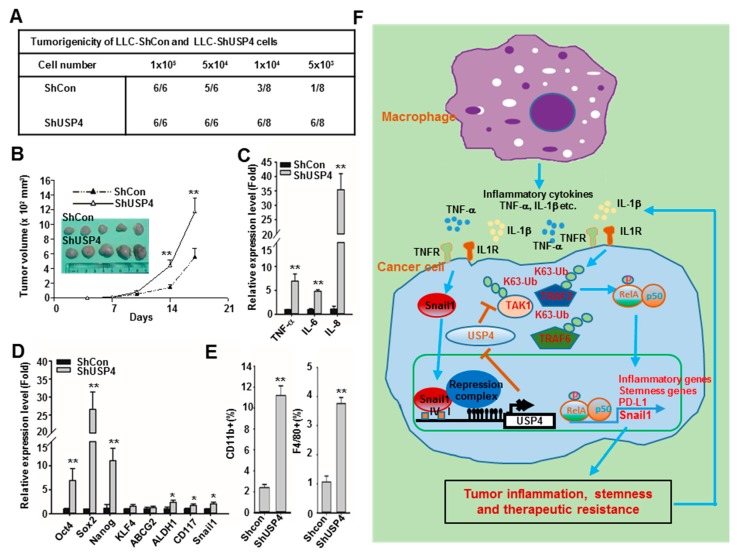
Downregulation of USP4 promotes tumorigenesis, tumor growth, tumor inflammation, and tumor stemness in mice. (**A**) C57BL/6 mice were subcutaneously (sc) injected with varying numbers of control and USP4 knockdown LLC cells. Number of tumors were counted at week six. (**B**) Mice were injected with 2 × 10^5^ cells. Tumor volume was measured at the indicated time points, and mean tumor size plotted (mean ± SD, n = 5). Mice were scarified on day 20 and tumors were photographed. (**C**,**D**) Expression of inflammatory cytokines (C) and stemness-associated genes (D) in tumors as analyzed by RT-qPCR. (**E**) Relative numbers of CD11b^+^ leukocytes and F4/80^+^ macrophages compared between tumors derived from control and USP4 knockdown cells by flow cytometry. Data presented as mean ± SD, n = 5. * *P* < 0.05; ** *P* < 0.01. (**F**) Illustration of a pro-tumor mechanism involving epigenetic silencing of USP4 by Snail1. Macrophages and inflammatory stimuli in the tumor microenvironment promote expression of Snail1 in cancer cells, which epigenetically suppresses the expression of USP4, leading to increased inflammation, stemness, and therapeutic resistance. The inflammatory cytokines released from cancer cells can further recruit macrophages into the tumor microenvironment and enhance expression of Snail1 to drive a positive feedback loop promoting tumor group.

The influences of USP4 expression levels on tumorigenesis and tumor growth were further investigated in mice inoculated with USP4 knockdown or control LLC cells. A higher tumor development rate and a faster growth rate was observed for tumors derived from USP4 knockdown cells (Figure 8A,B). These tumors also demonstrated higher expression levels of the inflammatory cytokines TNF-α, IL-6, and IL-8 as well as the stemness-associated genes Oct4, Sox2, Nanog, KLF4, ABCG2, ALDH1, CD117, and Snail1 compared to control cell-derived tumors (Figure 8C,D). In addition, flow cytometry analysis showed greater accumulation of CD11b+ leukocytes and F4/80+ macrophages in tumors derived from USP4 knockdown cells compared to control cell-derived tumors (Figure 8E). In line with these, depletion of macrophages with clodrondate liposomes inhibited tumor growh of LLC cells, reduced the expression of Snail1 and increased the expression of USP4 in tumors (Figure S4). 

In summary, both in vitro and in vivo findings in this study reveal a critical mechanism for regulation of lung tumorigenicity and aggression involving Snail1-mediated epigenetic downregulation of USP4 triggered in part by tumor associated macrophages. In turn, downregulation of USP4 in lung cancer cells further promotes inflammation, stemness, and therapeutic resistance of cancer cells (Figure 8F).

## 3. Discussion

Epigenetic regulation of gene transcription plays a crucial role in maintaining normal cellular homeostasis, while dysregulation of this process can result in the onset and progression of diseases including inflammation related cancers. Inflammation contributes to the initiation of epigenetic alteration and enhances the stemness of cancer cells. Conversely, epigenetic modification and inflammation are elevated in stemness-enriched cancer cells [23,24]. These observations indicate that the presence of a positive feedback loop driving tumorigenesis and tumor growth through regulation of epigenetics, inflammation, and stemness. Nevertheless, the molecular mechanism linking these processes is not well understood. In this study, we reveal a functional mechanism in which epigenetic suppression of the de-ubiquitinase USP4 by the transcription factor Snail1 activates a positive feedback loop driving increased inflammatory cytokine production, stemness, chemical resistance, and immune resistance of lung cancer cells, thereby accelerating tumor development.

In contrast to epigenetic regulation of gene expression, ubiquitination is a post-transcriptional modification that reduces protein expression through targeted proteolytic degradation and in addition can modulate protein−protein interactions for cell signaling [30,31]. By specifically disassembling ubiquitin chains, DUBs help control both protein expression levels and cell signaling. Moreover, dysregulation of de-ubiquitination by DUBs results in various clinical disorders [32,33]. In this study, OncoLnc screening for DUBs potentially involved in lung cancer development revealed that USP4 has the strongest negative Cox coefficient, suggesting a protective effect of expression against lung cancer. Further database analysis revealed an association between low USP4 expression and shorter patient survival. In tissue samples from lung cancer patients, decreased expression of USP4 was associated with advanced cancer stage. In line with these results, a protective effect of USP4 was reported in lung adenocarcinoma and breast cancer patients, although distinct effects of USP4 have been reported for other cancer types [48,49,50,51,52,53]. For instance, expression of USP4 was downregulated in lung adenocarcinoma, and low USP4 expression was associated with poor overall survival and recurrence-free survival [48]. Expression of USP4 was also significantly reduced in breast cancer tissue. Moreover, expression of this DUB inhibited cell proliferation in vitro and suppressed tumor growth in an animal model [49]. In contrast, increased expression of USP4 was found in tissue samples from hepatocellular carcinoma, melanoma, esophageal cancer, and colorectal cancer, and USP4 was shown to be oncogenic in these cancer cells [50,51,52,53]. These results suggest that the expression and tumor promoting effect of USP4 may be cancer type specific and that aberrant expression of USP4 can either increase or decrease tumorigenesis depend on the different cell contexts.

The low expression of USP4 in lung cancer cells and tumors results from Snail1-mediated epigenetic suppression. In stemness-enriched sphere cells, downregulation of USP4 was associated with upregulation of stemness-associated genes including Snail1. Expression of Snail1 was inversely correlated with the expression of USP4 in lung tumors. Snail1 binds to the E-box motif of the promoter of its target gene such as E-cadherin and epigenetically suppresses transcriptional expression. In this process, Snail1 recruits multiple chromatin enzymes to facilitate methylation at CpG-dinucleotides in GC rich regions of the promoter [25,26,27]. Analysis of the USP4 promoter region revealed multiple GC rich regions and E-box motifs required for Snail1 binding. In addition, overexpression of Snail1 promoted methylation of the USP4 promoter, suppressed promoter activity, and reduced USP4 expression in cultured lung cancer cells. Further, we found elevated DNA methylation of the USP4 promoter in lung tumor cells compared to normal tissue. Tumors with high Snail1 expression are usually more difficult to eradicate by therapeutic treatments, and the expression of Snail1 is frequently associated with poor prognosis. These effects of Snail1 involve regulation of its target genes, particularly suppression of those involving in cell adherence such as E-cadherin, occluding and claudins, which enhances cancer cell migration and invasion by increasing EMT [25,26,27,28,29]. The current study suggests that USP4 is a novel target mediating the tumorigenic effect of Snail1 by controlling the signaling for inflammation and stemness in cancer cells.

USP4 has been shown to modulate NF-κB dependent inflammatory responses through regulation of key components in inflammatory signaling pathways. USP4 regulates both IL-1β- and TNF-α-induced NF-κB activation through de-ubiquitination of TNF-receptor-associated factor (TRAF)2 and TRAF6, and this de-ubiquitination can inhibit IL-1β- and TNF-α-induced cancer cell migration [34]. In addition, USP4 was reported to block inflammatory responses induced by TLR− NF-κB and IL-1R− NF-κB signaling pathways by targeting TRAF6 and TAK1 [35,36]. USP4 knockout mice displayed enhancement of TAK1-NF-κB mediated liver inflammation in a hepatic ischaemia/reperfusion model [54]. Consistent with these findings, we found that USP4 knockdown in lung cancer cells increased phosphorylation of RelA, which is essential for NF-κB activation, and enhanced both basal and stimulus-induced inflammatory cytokine production, including production evoked by TNF-α and TLR ligands. In addition to acting as a master regulator of inflammation, NF-κB also regulates the stemness of cancer cells and promotes the shift to the CSC phenotype. Activated NF-κB can directly increase cancer cell stemness through transcriptional upregulation of stemness-associated genes, as well as indirectly through an autocrine pathway. For example, NF-κB activation in cancer cells by inflammatory stimuli results in the production of IL-6, which in turn activates expression of stemness-associated genes through a IL-6−JAK−STAT3 pathway [21,22,55,56]. In this study, UPS4 knockdown in multiple lung cancer cell lines increased the production of IL-6 as well as stemness properties. Thus, suppression of USP4 could increase cancer cell stemness both directly and indirectly through upregulated NF-κB activation.

The expression of Snail1 in cancer cells is regulated by multiple transcription factors and upstream signals, including NF-κB [25,26]. The tumor microenvironment contains various cell types aside from cancer cells, such as fibroblasts, endothelial cells, and leukocytes, most of which are macrophages [42,43]. In lung and other cancers, extensive macrophage infiltration is often associated with poor prognosis [5,6]. These tumor-associated macrophages (TAMs) release a wide variety of cytokines including TNF-α and IL-1β to activate NF-κB in cancer cells and promote Snail1 expression, inflammation, cancer stem cell niches, and all aspects of tumor progression [6,42]. Furthermore, a recent research suggested that NF-κB signaling in cancer cells elicited by macrophages results in expending of PD-L1 positive cancer cells and render these cells more resistence to conventional chemotherapy and cancer immunotherapy [57]. In line with these observations, this study showed that macrophages as well as the macrophage-derived pro-inflammatory cytokines TNF-α and IL-1β upregulated Snail1 expression in lung cancer cells, which in turn epigenetically suppressed USP4 expression. In addition to the increased inflammation and stemness, knockdown of USP4 in lung cancer cells also increased resistence to chemotherapy drugs and expressing with higher level of PD-L1. These findings suggest that epigenetic suppression of USP4 expression is a major functional mechanism underlying TAM-induced and microenvironment-induced Snail1 overexpression in lung cancer cells.

## 4. Materials and Methods

### 4.1. Reagents and Antibodies

Human recombinant tumor necrosis factor (TNF)-α, interleukin (IL)-1β, epidermal growth factor (EGF), and basic fibroblast growth factor (bFGF) were purchased from Peprotech (Rocky Hill, NJ, USA). Cisplatin, doxorubicin, BMS-345541, and anti-Flag M2 antibody were purchased from Sigma-Aldrich Co. (St. Louis, MO, USA). The TLR agonists Pam3Cys, polyI:C, LPS, and R848 were purchased from InvivoGen (San Diego, CA, USA) and CpG-ODNs was obtained from Invitrogen (Carlsbad, CA, USA). Antibodies against human and mouse USP4 were purchased from Gentex (Taipei, Taiwan) and Invitrogen, respectively, anti-PD-L1 from eBioscience/Thermo Fisher (Waltham, MA, USA), and rabbit anti-snail1, anti-phospho-RelA (Alexa Fluor 647 conjugate), and rabbit IgG isotype control antibody from Cell Signaling (Beverley, MA, USA). Reagents for MTS and luciferase assays were purchased from Promega (Madison, WI, USA). A human lung cancer tissue qPCR array (TissueScan Lung Cancer Tissue qPCR Panel III) was purchased from OriGene Technologies (Rockville, MD, USA).

### 4.2. Bioinformatics Analysis

The OncoLnc database (http://www.oncolnc.org) was searched for Cox coefficients between DUB gene expression levels and survival according to Kaplan-Meier estimates. Cox coefficients of different DUBs in lung adenocarcinoma were illustrated by heat maps generated using CIMminer software (https://discover.nci.nih.gov/cimminer/). Gene expression levels were compared between cancer stem-like cells and parental cancer cells by analyzing the GEO database. Expression fold-changes were analyzed using GEO2R (https://www.ncbi.nlm.nih.gov/geo/). The Oncomine database (https://www.oncomine.org/) was searched for gene expression profiles of normal tissues and tumors from patients. Putative Snail binding sites in the USP4 promoter (E-Box sites) were identified using Consite software (http://consite.genereg.net), while promoter GC rich (or CpG-rich) regions as potential methylation sites were identified using MethPrimer (http://www.urogene.org/cgi-bin/methprimer/methprimer.cgi).

### 4.3. Cell Culture and Macrophage Co-Culture Assays

Human embryonic kidney (HEK) 293 cells, human H1299 lung cancer cells, murine D121 lung cancer cells, and LLC lung cancer cells were grown in Dulbecco’s modified Eagle’s medium (DMEM) supplemented with 10% fetal bovine serum (FBS). Human H460 and HCC827 lung cancer cells were grown in RPMI medium supplemented with 10% FBS. BMDMs were isolated from 6- to 8-week-old C57BL/6J mice by culturing extracted bone marrow cells in a 7:3 mixture of DMEM and L929 conditioned medium supplemented with 10% FBS for 5 days. The BMDMs were then grown in DMEM supplemented with 10% FBS. For macrophage co-culture assays, LLC lung cancer cells were co-cultured with BMDMs in 0.4 μm transwell plates for 24 h with BMDMs on the upper chamber surface and cancer cells on the lower chamber surface.

### 4.4. Plasmid Construction

Various luciferase reporter genes under control of the wild type (WT) or mutant USP4 promoter were constructed to assess Snail1 binding and methylation sites. Briefly, a 969-bp USP4 promoter region spanning from −926 to +43 relative to the start codon was PCR amplified from genomic DNA of human H1299 lung cancer cells and subcloned into the pGL4.2 vector. DNA fragments containing the USP4 promoter with the E-box mutations shown in Table S4 were generated by two-step RT-PCR and subcloned into the pGL4.2 vector. A USP4 construct was generated through PCR amplification from human cDNA, followed by cloning into the pCDH plasmid. For knockdown of USP4 expression, shUSP4 plasmids were purchased from National Core Facility of RNA interference at Academia Sinica (Taipei, Taiwan).

### 4.5. Generation of Lentiviruses and Stably Transfection

Lentiviruses were generated by transfection of lentiviral vectors and packaging plasmids into HEK293T cells with PolyJet reagent (SignaGen Laboratories, Rockville, MD, USA). Viral supernatants were collected 48 h following transfection. Cancer cell lines were spin-infected by plating cells in 12-well plates in the presence of 8 μg/mL polybrene (Sigma-Aldrich Co.) and lentiviral supernatants followed by centrifugation at 1100× *g* for 30 min. The cells were subject to selection with puromycin (3 ng/mL) to obtain stable cell lines.

### 4.6. SDS-PAGE and Immunoblot Analysis

Whole-cell lysates were prepared in modified RIPA buffer (Millipore, Burlington MA, USA) containing 1x protease inhibitor cocktail (Roche, Basel, Switzerland). Total protein concentrations were measured using Bio-Rad protein assay dye regent (Bio-Rad, Hercules, CA, USA). Proteins were separated by SDS-PAGE and transferred onto PVDF membranes (Millipore). The membranes were probed with specific antibodies as indicated, and then incubated with appropriate horseradish peroxidase (HRP)-conjugated secondary antibody. Protein bands were visualized as an estimate of expression level using enhanced chemiluminescence (ECL) reagent (PerkinElmer, Waltham, MA, USA) and a UVP BioSpectrum Imaging System. Glyceraldehyde 3-phosphate dehydrogenase (GAPDH) or β-actin expression was also estimated as a gel loading control.

### 4.7. Flow Cytometry

For flow cytometric analysis, cells were suspended in PBS containing 2% FCS and incubated with PE-conjugated anti-PD-L1 antibody at 4 °C for 30 min. For intracellular staining of phospho-RelA, cells were fixed and permeabilized using a BD Cytofix/Cytoperm Kit (BD Bioscience, San Diego, CA, USA) for 20 min, washed and resuspensed in 1 × BD Perm/wash buffer, and incubated with anti-phospho-RelA for 30 min. After washing, cells were analyzed on a FACS Calibur flow cytometer with CellQuest software (Becton Dickinson, San Jose, CA, USA). For analysis of leukocytes in mouse tumors, excised tumor masses were minced and digested in PBS containing 0.5% BSA, 0.25% collagenase II, 0.25% collagenase IV, and 0.05% deoxyribonuclease for 30 min. The reaction was stopped by adding DMEM containing 10% FBS. The cell suspension was then strained through a 70-μM strainer and red blood cells removed using RBC lysis buffer (eBioscience). The remaining tumor cells were incubated with PE-conjugated F4/80 or CD11b antibody for 30 min and the populations of F4/80^+^ macrophages and CD11b^+^ leukocytes were analyzed by flow cytometry.

### 4.8. Enrichment of Sphere-Forming Cancer Cells

For enrichment of sphere-forming cells, parent cancer cells were cultured at the indicated density on Costar Ultra-Low Attachment plates (Corning, Corning, NY, USA) or six-well plates coated with poly (2-hydroxyethyl methacrylate) (polyHEMA; Sigma-Aldrich Co.) in defined medium consisting of serum-free DMEM/F12-K, 1x ITS solution (Sigma-Aldrich Co.), 20 ng/mL human recombinant EGF, and 20 ng/mL human recombinant bFGF. PolyHEMA-coated plates were prepared by adding 1 mL of a 10 mg/mL polyHEMA solution in 95% ethanol to the six-well plates and incubating overnight in a laminar flow hood at room temperature for air drying. 

### 4.9. Transfection and Luciferase Reporter Assays

For luciferase reporter assays, cells were seeded on 24-well plates and allowed to adhere overnight. Cells were then co-transfected with the indicated luciferase reporter and expression plasmids using polyethylenimine, and luciferase activities were measured as fold-induction relative to the control plasmid encoding the wild type promoter.

### 4.10. RT-qPCR Analysis of Gene Expression

Total RNAs were purified using TRIzol (Invitrogen) or a total RNA extraction kit (Favogen, Ping-Tung, Taiwan) according to the manufacturers’ instructions. First-strand cDNA was synthesized using the Super Script III first-strand synthesis system (Invitrogen) and oligo-dT primers for first-strand cDNA synthesis. Quantitative PCR was performed with gene-specific primers (Tables S5 and S6) using an ABI PRISM 7900HT sequence detection system (Applied Biosystems, Foster City, CA, USA) and KAPA SYBR Fast qPCR Kit (KK4605) for gene expression analysis. The expression of target mRNA was normalized to that of glyceraldehyde 3-phosphate dehydrogenase (GAPDH). 

### 4.11. DNA Methylation Assay

Genomic DNA was extracted from control H1299 cells and H1299 cells stably expressing Snail1 using the QIAamp DNA Mini kit (Qiagen, Hilden, Germany) and subjected to bisulfite modification using the EpiTect Bisulfite Kit (Qiagen) according to manufacture’s protocol. Bisulfate-treated DNA was examined for methylation status of CpG islands in the USP4 promoter region. Briefly, 2 μg of genomic DNA was incubated with Bisulfite Mix and DNA protection buffer. Bisulfite DNA conversion was performed using a thermal cycler, and the converted DNA were purified using EpiTech spin columns. The USP4 gene promoter was amplified from the bisulfite-modified DNA by PCR using the primers specific to USP4 gene CpG island listed in Table S7.

### 4.12. Animal Experiments

Animal experiments were approved by the Institutional Animal Care and Use Committee of the National Health Research Institutes, Taiwan. The approval number is NHRI-IACUC-107017A. C57BL/6J mice were maintained and handled in accordance with the stated guidelines. Control LLC cells or LLC cells with stable USP4 knockdown suspended in 100 μL PBS were subcutaneously injected into C57BL/6 mice at 6–8 weeks of age. These mice were monitored for tumor growth. Tumor volume was calculated using the following formula: Tumor volume (mm^3^) = (length × width^2^)/2.

### 4.13. Statistics Analysis

Data are expressed as mean ± SD. Three independent repeats were performed for each assay. Treatment group means were compared by independent samples Student’s t test. A *p* < 0.05 (two-tailed) was considered significant for all tests.

## 5. Conclusions

In summary, as illustrated in Figure 8F this study revealed a novel pro-tumor mechanism in which TAMs and inflammatory stimuli of the tumor microenvironment promote expression of Snail1 and concomitant Snail1-mediated epigenetic UPS4 downregulation in cancer cells. This in turn increases pro-inflammatory cytokine release, which recruits additional macrophages into the tumor microenvironment, further enhancing Snail1 expression and suppressing UPS4. This positive feedback loop drives the rapid proliferation, increased stemness, and chemoresistance characteristic of aggressive tumor cells. Snail1 inhibitors are under investigation [25]. Agents which can block this Snail1 and USP4 mediated positive feedback loop might be able to inhibit tumor progression and therapeutic resistance.

## Supplementary Materials

The following are available online at https://www.mdpi.com/2072-6694/12/1/148/s1, Figure S1: Low expression of USP4 in different cancer types, Figure S2: Stable USP4 knockdown enhances proliferation of lung cancer cells, Figure S3: Stable USP4 knockdown anchorage-independent growth of lung cancer cells, Figure S4: Investigate the effect of macrophages on the expression of USP4 and Snail1 in tumors and tumor growth, Figure S5: Whole blots for immunoblots, Table S1: Cox coefficients between survival and expression levels of 72 DUBs in lung adenocarcinoma patients, Table S2: Clinical data of patients with samples included in a lung cancer cDNA array, Table S3: Downregulation of USP4 in stemness-enriched and therapy-resistant cancer cells, Table S4:Wild type and mutated DNA sequences of E-boxes in the USP4 promoter region, Table S5:Sequences of forward and reverse primers employed for PCR amplification of human genes, Table S6: Sequences of forward and reverse primers employed for PCR amplification of mouse genes, Table S7: Sequences of forward and reverse primers employed for bisulfite PCR amplification of the human USP4 promoter.

## Abbreviations

**Abb.**	**Full Name**
USP4	Ubiquitin-specific peptidase 4;
DUB	Deubiquitinase
NF-κB	Nuclear factor-κB
TLR	Toll-like receptor
IL	Interleukin
TNF-α	Tumor necrosis factor-α
TNFR	Tumor necrosis factor receptor
TRAF	TNFR-associated factor
MyD88	Myeloid differentiation primary response 88
IRAK	Interleukin-1 receptor-associated kinase
TAK	TGF-β-activated kinase
TRADD	Tumor necrosis factor receptor type 1-associated death domain protein
RIP	Receptor interacting protein
EMT	Epithelial−mesenchymal transition
CSC	Cancer stem-like cells
ELISA	Enzyme-linked immunosorbent assay
HEK	Human embryonic kidney
FCS	Fetal calf serum
PBS	Phosphate buffer saline
GAPDH	Glyceraldehyde 3-phosphate dehydrogenase
