# Supplementary material for: Epigenetic Silencing of Ubiquitin Specific Protease 4 by Snail1 Contributes to Macrophage-Dependent Inflammation and Therapeutic Resistance in Lung Cancer
Source: Cancers (Basel). 2020 Jan 8;12(1):148. doi: 10.3390/cancers12010148 (PMC7016945; doi:10.3390/cancers12010148)
Supplement: Supplementary file 1 [file cancers-12-00148-s001.pdf]

cancers

MDPI

Epigenetic Silencing of Ubiquitin Specific Protease 4 by Snail1 Contributes to Macrophage-Dependent Inflammation and Therapeutic Resistance in Lung Cancer

Chao-Yang Lai, Da-Wei Yeh, Chih-Hao Lu, Yi-Ling Liu, Yu-Chen Chuang, Jhen-Wei Ruan, Cheng-Yuan Kao, Li-Rung Huang, Tsung-Hsien Chuang

Content: Supplementary Tables S1-S7; Supplementary Figure S1-S5

Table S1. Cox coefficients between survival and expression levels of 72 DUBs in lung adenocarcinoma patients

DUB names	Cox Coefficient	DUB names	Cox Coefficient	DUB names	Cox Coefficient
USPs		USPs		OTUs	
USP1	0.121	USP31	0.009	OTUB1	0.111
USP2	-0.133	USP32	0.021	OTUB2	0.037
USP3	-0.040	USP33	-0.103	OTUD1	-0.117
USP4*	-0.345	USP34	-0.034	OTUD3	-0.148
USP5*	0.200	USP39	0.060	OTUD4	0.029
USP6	-0.111	USP40	0.122	OTUD5*	-0.152
USP7	0.031	USP42	0.053	OTUD7A	-0.117
USP8	-0.039	USP43	0.035	OTUD7B	0.023
USP9X	0.053	USP44*	-0.261	TRABID	-0.066
USP10	0.105	USP45	0.039	VCPIP1	0.066
USP11	-0.036	USP46	0.019	A20	0.059
USP12	-0.076	USP47	0.001	Josephin	
USP13	0.069	USP48	-0.133	ATXN3	-0.081
USP14*	0.217	USP49	-0.109	JOSD1*	0.217
USP15	0.025	USP51	-0.134	JOSD2	0.068
USP16	-0.090	USP52*	-0.181	JAMM/MPN	
USP18	0.138	USP53	-0.004	BRCC36	0.046
USP19*	-0.177	USP54	-0.136	CSN5	0.101
USP20*	-0.218	USPL1	-0.098	POH1*	0.205
USP21	-0.035	CYLD*	-0.209	AMSH	0.127
USP22	0.043	UCHs		AMSH-LP	0.126
USP24	-0.101	UCHL1	0.092	MPND*	-0.218
USP25	0.053	UCHL3	0.094	MYSM1	-0.103
USP27X	-0.118	UCHL5*	0.228	PRPF8	-0.031
USP28	-0.019	BAP1*	-0.152	*: p < 0.05	
USP30	-0.124				

Table S2. Clinical data of patients with samples included in a lung cancer cDNA array.

Sample number	W ell position	Appearance	SAMPLE diagnosis from pathology verification	Tumor Grade	Minimum Stage Grouping	Normal (%)	Lesion (%)	Tumor (%)
1	C01	Normal	Within normal limits	Not Applicable	Not Applicable	100	0	0
2	C02	Normal	Within normal limits	Not Applicable	Not Applicable	100	0	0
3	C03	Normal	Within normal limits	Not Applicable	Not Applicable	100	0	0
4	C04	Normal	Within normal limits	Not Applicable	Not Applicable	100	0	0
5	C05	Normal	Within normal limits	Not Applicable	Not Applicable	100	0	0
6	C06	Normal	Within normal limits	Not Applicable	Not Applicable	100	0	0
7	C07	Normal	Within normal limits	Not Applicable	Not Applicable	100	0	0
8	C08	Normal	Within normal limits	Not Applicable	Not Applicable	100	0	0
9	C09	Tumor	Carcinoma of lung, squamous cell	AJCC G2: Moderately differentiated	IA	10	0	70
10	C10	Tumor	Carcinoma of lung, large cell	Not Reported	IA	0	0	90
11	C11	Tumor	Adenocarcinoma of lung	AJCC G2: Moderately differentiated	IA	0	0	70
12	C12	Tumor	Carcinoma of lung, bronchioloalveolar, non-mucinous	Not Reported	IA	0	0	98
13	D01	Tumor	Adenocarcinoma of lung	AJCC G1: Well differentiated	IA	10	0	70
14	D02	Tumor	Adenocarcinoma of lung	AJCC G2: Moderately differentiated	IA	0	0	80
15	D03	Tumor	Carcinoma of lung, squamous cell	Not Reported	IB	10	0	80
16	D04	Tumor	Carcinoma of lung, squamous cell	AJCC G2: Moderately differentiated	IB	0	0	55
17	D05	Tumor	Carcinoma of lung, squamous cell	AJCC G3: Poorly differentiated	IB	0	0	55
18	D06	Tumor	Adenocarcinoma of lung, bronchioloalveolar	Not Reported	IB	5	0	60
19	D07	Tumor	Carcinoma of lung, neuroendocrine	Not Reported	IB	0	0	70
20	D08	Tumor	Carcinoma of lung, squamous cell	AJCC G3: Poorly differentiated	IB	10	0	60
21	D09	Tumor	Adenocarcinoma of lung	AJCC G3: Poorly differentiated	IIA	1	0	80
22	D10	Tumor	Carcinoma of lung, small cell, metastatic	AJCC G3: Poorly differentiated	IIA	0	0	50
23	D11	Tumor	Carcinoma of lung, squamous cell	AJCC G3: Poorly differentiated	IIA	10	0	80
24	D12	Tumor	Adenocarcinoma of lung	AJCC G2: Moderately differentiated	IIB	0	0	60
25	E01	Tumor	Carcinoma of lung, squamous cell	Not Reported	IIB	0	0	75
26	E02	Tumor	Adenocarcinoma of lung	AJCC G2: Moderately differentiated	IIB	0	0	75
27	E03	Tumor	Carcinoma of lung, squamous cell	AJCC G3: Poorly differentiated	IIB	25	0	65
28	E04	Tumor	Adenocarcinoma of lung	AJCC G3: Poorly differentiated	IIB	10	0	80
29	E05	Tumor	Adenocarcinoma of lung	AJCC G3: Poorly differentiated	IIB	0	0	90
30	E06	Tumor	Carcinoma of lung, squamous cell	AJCC G3: Poorly differentiated	IIB	10	0	65
31	E07	Tumor	Carcinoma of lung, squamous cell	Not Reported	IIB	0	0	60
32	E08	Tumor	Carcinoma of lung, squamous cell	AJCC G3: Poorly differentiated	IIIA	5	0	40
33	E09	Tumor	Carcinoma of lung, adenosquamous	Not Reported	IIIA	0	0	70
34	E10	Tumor	Carcinoma of lung, large cell, neuroendocrine	AJCC G3: Poorly differentiated	IIIA	0	0	50
35	E11	Tumor	Carcinoma of lung, small cell	Not Reported	IIIA	5	0	50
36	E12	Tumor	Adenocarcinoma of lung	AJCC G2: Moderately differentiated	IIIA	0	0	74
37	F01	Tumor	Carcinoma of lung, sarcomatoid	AJCC G3: Poorly differentiated	IIIB	0	0	55
38	F02	Tumor	Adenocarcinoma of lung, bronchioloalveolar	Not Reported	IIIB	5	0	90
39	F03	Tumor	Carcinoma of lung, adenosquamous	AJCC G3: Poorly differentiated	IIIB	40	0	50
40	F04	Tumor	Adenocarcinoma of lung, bronchioloalveolar	AJCC G1: W ell differentiated	IIIB	5	0	50
41	F05	Tumor	Carcinoma of lung, squamous cell	AJCC G3: Poorly differentiated	IIIB	0	0	85
42	F06	Tumor	Adenocarcinoma of lung	AJCC G2: Moderately differentiated	IV	10	0	40
43	F07	Tumor	Carcinoma of lung, non-small cell, metastatic	Not Reported	IV	10	0	85
44	F08	Tumor	Carcinoma of lung, non-small cell, metastatic	AJCC G3: Poorly differentiated	IV	25	0	60
45	F09	Tumor	Carcinoma of lung, squamous cell, metastatic	AJCC G3: Poorly differentiated	IV	0	0	90
46	F10	Tumor	Adenocarcinoma of lung	AJCC G2: Moderately differentiated	IV	10	0	70
47	F11	Tumor	Adenocarcinoma of lung, metastatic	AJCC G3: Poorly differentiated	IV	0	0	80
48	F12	Tumor	Adenocarcinoma of lung, metastatic	Not Reported	IV	0	0	80

Table S3. Downregulation of USP4 in stemness-enriched and therapy-resistant cancer cells.

Down-regulation of USP4 (> 2-fold) in cancer stem-like cells			
	Cell type	Log FC	Accession no.
HNSCC	SAS sphere	-1.322	GSE35603
	FaDu Bmi1 overexpression	-1.024	GSE35603
Breast cancer	MCF-7 snail1 overexpression	-1.862	GSE35603
	MCF-7 doxorubucin resistant	-1.230	GSE24460
Lung cancer	PC9 gefitinib resistant	-1.080	GSE60189
	A549 radioresistant	-1.488	GSE35603

Table S4. Wild type and mutated DNA sequences of E-boxes in the USP4 promoter region.

	Promoter region	Sequence
E-box		CANNTG
E-box-I	-458 to -453	CACCTG
mutant E-box-I	-458 to -453	AACCTA
E-box-II	-516 to -511	CAGCTG
mutant E-box-II	-516 to -511	AAGCTA
E-box-III	-538 to -533	CAGCTG
mutant E-box-III	-538 to -533	AAGCTA
E-box-IV	-684 to -679	CAGGTG
mutant E-box-IV	-684 to -679	AAGGTA

Table S5. Sequences of forward and reverse primers employed for PCR amplification of human genes.

USP4	Forward 5'-CTTATTGACAGCCGGTGGTT-3'
	Reverse 5'-GTTTATTCCACGCCTCGGTA-3'
GAPDH	forward 5'-GAGTCAACGGATTTGGTCGT-3'
	reverse 5'-GACAAGCTTCCCGTTCTCAG-3'
OCT-4	forward 5'-GAAGGATGTGGTCCGAGTGT-3'
	reverse 5'-GTGAAGTGAGGGCTCCCATA-3'
SOX-2	forward 5'-ACACCAATCCCATCCACACT-3'
	reverse 5'-GCAAACTTCCTGCAAAGCTC-3'
NANOG	forward 5'-TTCCTTCCTCCATGGATCTG-3'
	reverse 5'-ATCTGCTGGAGGCTGAGGTA-3'
KLF-4	forward 5'-ACCCACACAGGTGAGAAACC-3'
	reverse 5'-ATGTGTAAGGCGAGGTGGTC-3'
ABCG2	forward 5'-GTGGCCTTGGCTTGTATGAT-3'
	reverse 5'-AACAATTGCTGCTGTGCAAC-3'
ALDH1	forward 5'-TGTTAGCTGATGCCGACTTG-3'
	reverse 5'-CTTCTTAGCCCGCTCAACAC-3'
TNF-α	forward 5'-AACCTCCTCTCTGCCATCAA-3'
	reverse 5'-CCAAAGTAGACCTGCCCAGA-3'
IL-1β	forward 5'-ACGATGCACCTGTACGATCA-3'
	reverse 5'-TCTTTCAACACGCAGGACAG-3'
IL-6	forward 5'-TACCCCCAGGAGAAGATTCC-3'
	reverse 5'-TTTTCTGCCAGTGCCTCTTT-3'
IL-8	forward 5'-GTGCAGTTTTGCCAAGGAGT-3'
	reverse 5'-CTCTGCACCCAGTTTTCCTT-3'
SNAIL1	forward 5'-TTTACCTTCCAGCAGCCCTA-3'
	reverse 5'-CCCACTGTCCTCATCTGACA-3'

Table S6. Sequences of forward and reverse primers employed for PCR amplification of mouse genes.

Usp4	Forward 5'-TCACCTCAAGCGTTTCTCCT-3'
	Reverse 5'-CCATTTCCCGTTCAGTCTGT-3
Gapdh	forward 5'-ACCCAGAAGACTGTGGATGG-3'
	reverse 5'-CACATTGGGGGTAGGAACAC-3'
Oct-4	forward 5'-AAGCCCTCCCTACAGCAGAT-3'
	reverse 5'-CTGGGAAAGGTGTCCCTGTA-3'
Sox-2	forward 5'-AAGGGTTCTTGCTGGGTTTT-3'
	reverse 5'-AGACCACGAAAACGGTCTTG-3'
Nanog	forward 5'-CCAGTGGAGTATCCCAGCAT-3'
	reverse 5'-GAAGTTATGGAGCGGAGCAG-3'
Klf-4	forward 5'-GCAGTCACAAGTCCCCTCTC-3'
	reverse 5'-CTGTGTGAGTTCGCAGGTGT-3'
Abcg2	forward 5'-CCATTCATCAGCCTCGGTAT-3'
	reverse 5'-AATCCGCAGGGTTGTTGTAG-3'
Aldh1	forward 5'-GCACTCAATGGTGGGAAAGT-3'
	reverse 5'-TTTGGCCACACACTCCAATA-3'
Tnf-α	forward 5'-AGCCCCCAGTCTGTATCCTT-3'
	reverse 5'-CTCCCTTTGCAGAACTCAGG-3'
Il-1β	forward 5'-CAGGCAGGCAGTATCACTCA-3'
	reverse 5'-AGCTCATATGGGTCCGACAG-3'
Il-6	forward 5'-AGTTGCCTTCTTGGGACTGA-3'
	reverse 5'-TCCACGATTTCCCAGAGAAC-3'
Il-8	forward 5'-CGTCCCTGTGACACTCAAGA-3'
	reverse 5'-TAATTGGGCCAACAGTAGCC-3'
Snail1	forward 5'-TTCACCTTCCAGCAGCCCTA-3'
	reverse 5'-GCCACTGTCCTCATCGGACA-3'

Table S7. Sequences of forward and reverse primers employed for bisulfite PCR amplification of the human USP4 promoter.

Usp4 promoter	Forward 5'-TTTGGTTTGTATTTATTTAGGATTA-3'
	Reverse 5'-ATCCTCATTAAAAATCCAAACTC-3

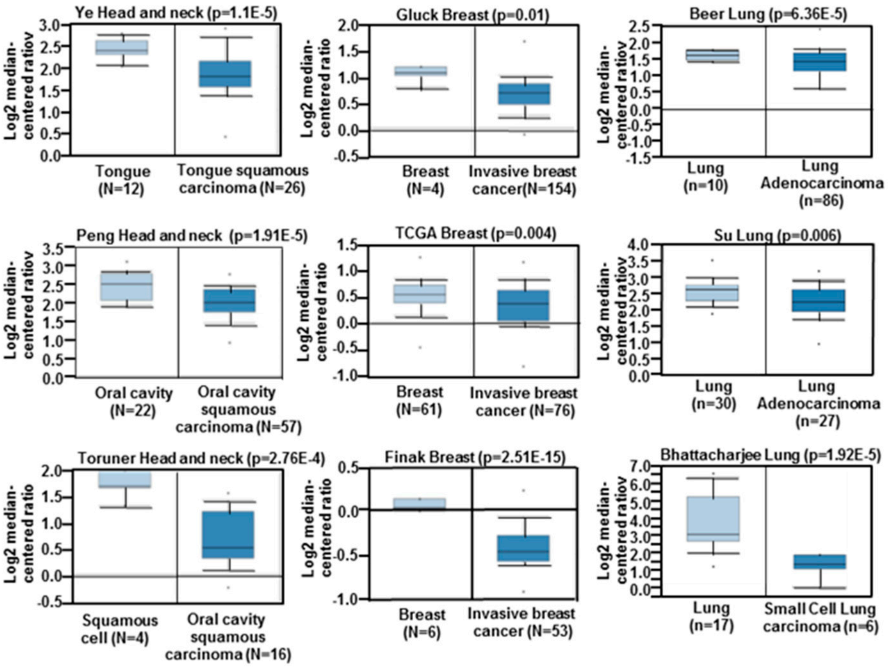

Supplementary Figure S1. Low expression of USP4 in different cancer types. Data were acquired from the Oncomine database. Tissue sample numbers are shown in parentheses.

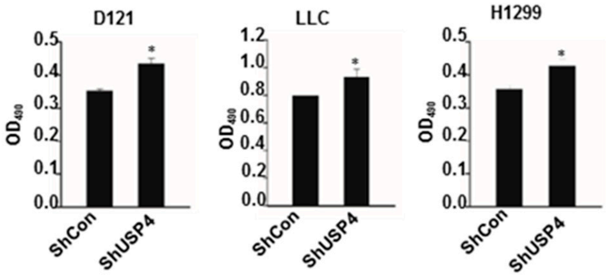

Supplementary Figure S2. Stable USP4 knockdown enhances proliferation of lung cancer cells. Cells were seed onto 96-well plates at 5000 cells/well for 48 h and then incubated with CellTiter 96 AQueous Non-Radioactive Cell Proliferation (MTS) reagent (Promega) for 2 h. The absorbance at 490 nm was measured using an Envision Alpha Multilabel Reader (PerkinElmer). Data presented mean ± SD. *P < 0.05.

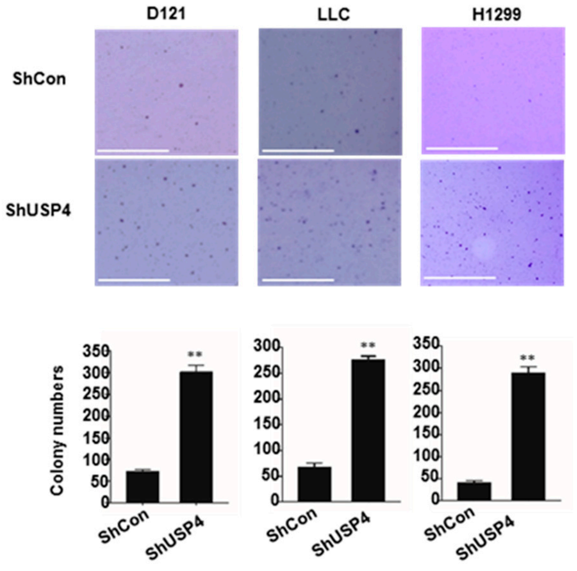

Supplementary Figure S3. Stable USP4 knockdown anchorage-independent growth of lung cancer cells. For anchorage-independent colony forming assays, bottom agar (DMEM, 10% FBS, 0.8% agarose) was added to each well of six-well tissue culture plates and allowed to solidify. Top

agar (DMEM, 10% FBS, 0.4% agarose) containing cells (1×10^4/well) was added, followed by the addition of growth medium. The plates were incubated at 37 °C for 2−3 weeks and colony formation was monitored under light microscopy. The plates were fixed with 4% formaldehyde and stained with 0.005% crystal violet in PBS for 1 h, and colonies were photographed and counted. The total number of colonies with a diameter of ≥100 µm was quantified over four randomly chosen fields per well from triplicate repeats (12 fields per condition). Colonies were counted after 3 weeks. Top panels: Representative photographs showing colony formation. Bottom bar figures: enumeration of colonies in three culture wells. White scale bar: 1 cm. Data presented mean ± SD. *P < 0.05; **P < 0.01.

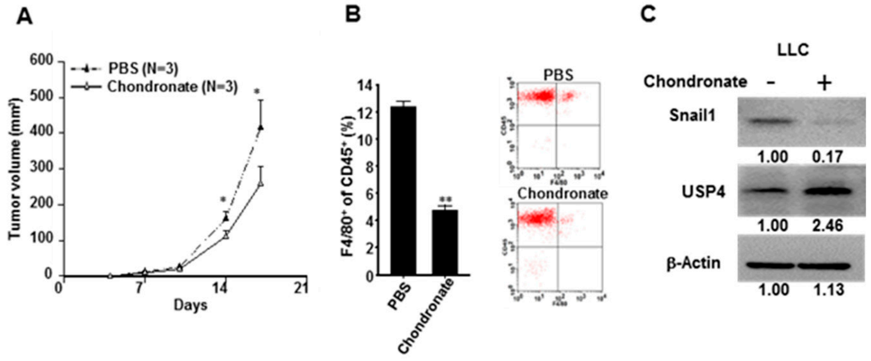

Supplementary Figure S4. To investigate the effect of macrophages on the expression of USP4 and Snail1 in tumors and tumor growth, C57BL/6 mice were injected with or without clodronate liposomes for macrophage depletion with 100 µl of clodronate liposomes 2 days before inoculation of cancer cells and then every 5 days. These micewere subcutaneously injected with 2×10^5 of LLC cells in 100 µl of PBS. (A) These mice were monitered for tumor growth. The mice were scarified at day 20. (B) Populations of F4/80+, CD45+ macrophage in blood samples were analyzed with flow cytometry. Left panel shows a set of histograms for the flow analysis. (C) Expression of Snail1 and USP4 in the tumors are detected with immunoblotting. β-actin was blotted as control. Data presented mean ± SD. *P < 0.05; **P < 0.01.

Figure 3G

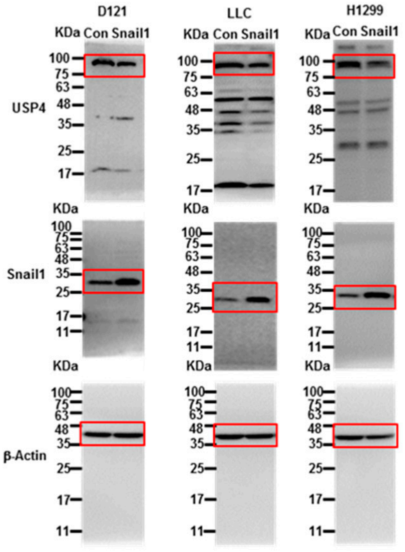

Figure 6A

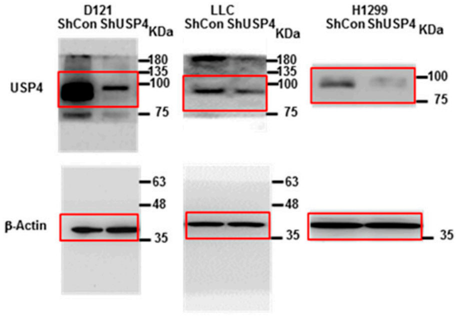

Figure 5F

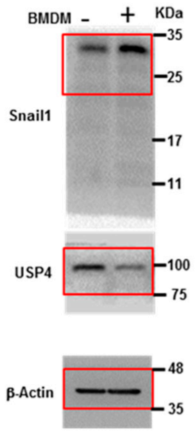

Figure 5G

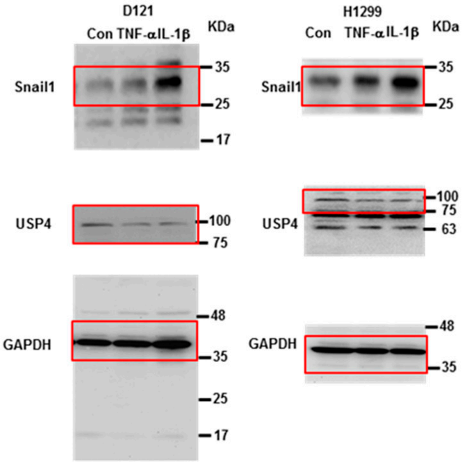

Supplementary Figure S5. Whole blots for immunoblots shown in Figure 3G, Figure 4F and G, and Figure 5A
